# Editorial: Multi-omics strategies to analyze complex agronomic traits in plants

**DOI:** 10.3389/fpls.2023.1256629

**Published:** 2023-07-31

**Authors:** Lin Chen, Guo-Fei Tan

**Affiliations:** ^1^ Institute of Animal Science, Chinese Academy of Agricultural Sciences, Beijing, China; ^2^ Institute of Horticulture, Guizhou Academy of Agricultural Sciences (GAAS), Guiyang, Guizhou, China

**Keywords:** omics, genetic selection, complex traits, genetic improvement, crop breeding

Complex traits in plants are usually controlled by multiple genes, and the genetic analysis of complex traits has always been a difficult problem in plant research. In the past decade, with the continuous development of omics technology, more and more omics technologies (genomics, transcriptome, metabolomics, proteomics, and others) have been applied to the genetic analysis of plant complex traits. Therefore, how to integrate these massive omics data has become a new research hotspot.

In the past year, a total of 21 manuscripts focusing on this topic were received, and 9 original research articles were selected for publication after rigorous peer review. These 9 articles reported research on seven different plants, namely, maize, celery, lettuce, apple, rapeseed, sweet cherry, and bamboo shoot. Among these articles, there are seven research articles focusing on the agronomic traits related to plant development, one research article focusing on heat stress related to plant response to abiotic stress, and one research article focusing on plant response to biotic stress. To the benefit of potential readers, the key points of the nine articles in this Research Topic are highlighted as follows:

The fleshy stem is the main product organ of stem lettuce. However, the molecular mechanism of the fleshy stem expansion in lettuce is still unknown. Huang et al. identified the key genes and metabolites during the fleshy stem expansion process by a comparative analysis of the transcriptome and metabolome. A total of 9,383 differentially expressed genes and 822 metabolites were found during this process. Moreover, these differentially expressed genes and metabolites were significantly enriched in the sugar synthesis, glycolysis, and plant hormone processes. These results provide important candidate genes and metabolites for further research into the molecular mechanisms of fleshy stem expansion.

The rapid decline in the quality of fresh bamboo shoots after receipt is one of the main reasons for the difficulty of their long-distance transportation and long-term preservation. Li, Z. et al. conducted morphological, physiological, transcriptomic, and microRNA-sequencing analyses to investigate the postharvest characteristics of fresh bamboo shoots. The differentially expressed genes and miRNAs were significantly enriched in structural polysaccharide metabolism, starch and sucrose metabolism, and glycolysis pathways. Furthermore, a co-expression network of carbohydrate metabolism was constructed in this article. These results suggest that carbohydrate metabolism mainly affects the quality of postharvest bamboo shoots.

Gibberellin (GA) is an important hormone in the plant development process. Exogenous use of GA3 can improve the sweet cherry yield but decrease the fruit quality. Chen et al. identified the key genes and metabolites by integrating transcriptome and metabolome analysis in response to exogenous GA3 application in sweet cherry. They results showed that the content of ABA, JA, and IAA were significantly increased after the application of exogenous use of GA3. Moreover, they also found the WRKY transcription factors were more sensitive to the application of exogenous GA3. Their results provide a new insight into improving the yield and quality of sweet cherry.

Flowering time is one of the most important traits of maize and other plants for breeding. Wu et al. identified 82 significant SNPs and 117 candidate genes related to the maize flowering time by the GWAS method with an association panel consisting of 226 maize inbred lines. In addition, a total of 21 QTLs and 65 candidate genes were found to be related to maize flowering time by linkage analysis with an F2:3 population. By integrating GWAS, linkage analysis, and transcriptome analysis, 25 important candidate genes for maize flowering time were identified in this study. Their finding is novel and provides new gene resources for other researchers to study the genetic bases of maize flowering time.

Seminal roots play a key role for maize seedlings to obtain water and other nutrients. In this topic, Wang et al. identified three important candidate genes (Zm00001d021572, Zm00001d021579, and Zm00001d021861) by combining QTL analysis, which was conducted by using a maize–teosinte BC2F6 population, with transcriptome analysis. Moreover, they also found that Zm00001d021572 was selected during maize domestication. These results remind other researchers of the existence of excellent genetic resources in the wild germplasm resources of crops.

Plant height is the key element of the ideal plant architecture. The association mapping panel with 230 rapeseed accessions was used by Zhao et al. to understand the genetic basis of plant height in rapeseed. By integrating GWAS, haplotype analysis, and expression analysis, *BnaA01g09530D*, which encodes BRASSINOSTEROID-INSENSITIVE 2 and belongs to the GLYCOGEN SYNTHASE KINASE 3 (GSK3) family, was found to be significantly related to plant height in rapeseed. The results of this article provide an important candidate gene for the genetic improvement of rapeseed in line with the ideal plant architecture.

Besides plant height, the first branch height in rapeseed, which is another key element of the ideal plant architecture, has an important effect on yield and mechanized harvesting. Dong et al. found that 19 QTLs were related to the first branch height by linkage analysis, and 26 significant SNPs were found by GWAS. Among these hotspot regions, one major QTL located on Chr.A02 was found by GWAS and linkage analysis. In this region, it was confirmed by expression analysis and transgene analysis that *BnaA02g13010D*, which encodes a TCP transcription factor, can regulate the first branch height in rapeseed. These results provide valuable information for the genetic improvement of the first branch height in rapeseed.

Celery is a valuable edible crop and cannot endure high temperatures. Heat shock transcription factors (HSFs) are the most important transcription factors in plant response to heat stress and other abiotic stresses. Li, M. et al. identified 29 *AgHSFs* in celery and classified these genes into three classes. Based on the transcriptome data, the expression patterns of these genes were analyzed under heat stress. Among these genes, *AgHSFa6-1* acts as a positive regulator to improve the celery thermotolerance. These results provide a valuable gene resource for the improvement of celery to heat stress.

Alternaria blotch disease is one of the major fungal diseases in apple, which is caused by the *Alternaria alternata apple pathotype* (AAAP). To understand the molecular mechanism to AAAP in different varieties in apple, Liang et al. conducted allele-specific expression analysis and comparative transcriptomic analysis before and after AAAP inoculation. Based on this analysis, *MdFLS2* and its allele were found to be significantly related to resistance to AAAP in apple.

Based on the abovementioned articles published in this Research Topic, it can be seen that multi-omics techniques have greatly improved the efficiency of the genetic analysis of complex agronomic traits in plants, especially for some non-model plants. We hope that these research results can provide new ideas and assistance for the analysis of complex agronomic traits in plants. Finally, we are very grateful for the efforts of the journal editors, peer reviewers, and relevant authors. Without their efforts, this Research Topic would not appear in front of readers. We hope that readers can obtain valuable information from this topic to help them achieve success in the future.

## Author contributions

LC: Formal Analysis, Funding acquisition, Validation, Writing – original draft, Writing – review & editing. G-FT: Validation, Writing – original draft, Writing – review & editing.

